# Impact of gluteus maximus–focused intervention using Powers’ program on chronic mechanical low back pain: A randomized controlled trial

**DOI:** 10.1186/s13102-025-01475-x

**Published:** 2026-01-20

**Authors:** Alshaymaa Abdeldaiem Abdeldaiem, Enas Fawzy Youssef, Nesreen Fawzy Mahmoud, Mohamed Abdelmegeed

**Affiliations:** 1 Department of Physical Therapy for Musculoskeletal Disorders and its Surgery, Faculty of Physical Therapy, Horus University, New Damietta, Egypt; 2 Department of Orthopedic Physical Therapy, Faculty of Physical Therapy, Cairo University, Giza, Egypt; 3 Department of Allied Health and Kinesiology, School of Health Sciences, Hofstra University, 222E Hagedorn Hall, Hempstead, NY 11549-1190 USA

**Keywords:** Powers’ program, Chronic mechanical low back pain, Gluteus maximus, Hip muscle strengthening

## Abstract

**Background:**

Chronic mechanical low back pain (CMLBP) is frequently associated with poor neuromuscular control and decreased gluteus maximus (GM) muscle activation. Rather than being caused by direct muscle damage, these deficiencies are frequently the result of disrupted communication between ascending and descending motor pathways, resulting in dysfunctional lumbopelvic stabilizer activation patterns.

**Objective:**

The purpose of this study was to investigate the effect of GM activation, strengthening, and functional exercise using Powers’ program on pain, disability, GM strength, and hop test performance in subjects with CMLBP.

**Methods:**

A single-blinded, two parallel groups, randomized controlled clinical trial was conducted. Forty-eight subjects with CMLBP, with a mean age of (32.77 ± 6.10) were randomly allocated to either study or control groups. The study group received eight phases of the Powers’ program plus a conventional intervention of flexibility exercises, hot packs, and transcutaneous electrical stimulation (three sessions per week for four weeks). The active control group received the same conventional intervention only. All subjects were assessed pre- and post-intervention for pain, disability, GM strength, and functional performance using the Visual Analogue Scale, Oswestry Disability Index, Hand-held dynamometer, and one-legged hop tests (single-limb triple hop test for distance and single-limb crossover hop test for distance), respectively.

**Results:**

All outcomes showed significant within-group improvements (*p* < 0.05). The post-treatment between-group MANOVA comparison was not statistically significant (*p* > 0.05). Post-hoc univariate analysis, however, revealed an improvement in GM strength in the study group (*p* < 0.05). Nonetheless, this data should be interpreted with caution as the overall MANOVA was not significant, and repeated comparisons may increase the chance of a type I error.

**Conclusion:**

The eight-phase Powers program improved GM strength in subjects with CMLBP, demonstrating its effectiveness in targeted muscle strengthening. Non-significant findings, however, in pain, disability, and functional performance indicate that future research should look at lengthier therapies or integration with broader rehabilitation modalities to obtain functional benefits beyond strength improvement.

**Trial registration:**

The study was registered on ClinicalTrials.gov: NCT05770986 and the date of registration was March 16th, 2023.

**Supplementary Information:**

The online version contains supplementary material available at 10.1186/s13102-025-01475-x.

## Introduction

Chronic Mechanical low back pain (CMLBP) is a common musculoskeletal disorder that affects up to 20% of the worldwide population and 53% of the Egyptian population [1–4]. This type of back pain is a major cause of disability worldwide, with significant social and economic costs for its management [5].

CMLBP is defined as pain that originates in the spine, intervertebral discs, or adjacent soft tissues. It is usually linked with recurrent mechanical loads, altered movement patterns, and decreased muscle activation rather than structural disease. These mechanical dysfunctions contribute to poor lumbopelvic control and ongoing discomfort. Among the lumbopelvic muscles, the gluteus maximus (GM) plays a vital function in stabilizing the pelvis and regulating hip extension; inadequate or delayed activation of this muscle has been associated with movement compensation and higher spinal stress in subjects with CMLBP [3, 4].

The GM muscle acts as a powerhouse for core stability and movement control. Its anatomical connections, including the erector spinae and thoraco-lumbar fascia, allow it to stabilize the lower back and sacroiliac joint (SIJ). Additionally, co-contraction with the psoas major helps maintain stability in the lumbo-sacral region. This coordinated action allows the GM to control trunk and pelvic motion in all three planes (frontal, sagittal, and transverse) [6, 7]. It also provides the base of support necessary for the core muscles [8]. Moreover, during gait, the GM controls hip flexion during heel strike and generates hip extension torque during early stance. It has a limited contribution to propulsion but is necessary for supporting the trunk and pelvis on the femur. The GM acts in tandem with the hamstrings to resist external flexion moments and maintain an upright posture during weight acceptance phase [9].

Individuals with CMLBP frequently use compensatory movement patterns to reduce pain, such as reduced hip extension, increased lumbar extension, and overactivation of the hamstrings or lumbar erector spinae. These changes diminish GM recruitment and disrupt the usual lumbopelvic rhythm. A weakening GM can increase shear and compressive pressures across the lumbar spine, sacroiliac joint, and hip, resulting in poor load transfer and chronic dysfunction [10–12].

Other mechanical intervention, such as manual therapy or traction, have been employed in the treatment of low back pain [13], but the current study focused on an active exercise strategy. The Powers’ Program was chosen because it focuses on gluteus maximus activation and lumbopelvic motor control, which address neuromuscular weaknesses typically linked with persistent mechanical low back pain.

Previous research has shown that subjects with CMLBP have lower GM activation and morphological alterations [14, 15], hence strengthening this muscle is thought to be critical for restoring correct lumbopelvic rhythm and enhancing mechanical load transfer. Although these investigations focused on muscle activation rather than direct biomechanical forces, their results support the mechanical cause for targeted GM strengthening in CMLBP. This approach is well supported by Grade A recommendation of clinical practice guidelines which stated that “Physical therapists should use movement control exercise and specific trunk muscle activation for patients with CMLBP and movement control impairment” [16].

The gluteus maximus is a versatile hip muscle, acting as a primary extensor (particularly the posterior and superior fibers), an external rotator, and a secondary hip abductor. This multifunctional role suggests that exercises that target these various functions simultaneously might lead to a more robust activation of the GM. The Powers’ program, developed by Chris Powers, exemplifies this principle. It incorporates exercises that combine hip abduction, extension, and external rotation, progressing from less to more challenging variations across eight levels [17–20].

Previous research has shown that the Powers’ Program enhances GM activation and neuromuscular control during functional weight-bearing exercises in both athletes and healthy people [21, 22]. Although its use has not been studied in individuals with chronic mechanical low back pain, the underlying mechanical principles of this program—targeted GM strengthening, improved lumbopelvic stability, and restoration of proper movement mechanics—support its potential application in this population. Therefore, this study investigated whether the Powers’ program can enhance outcomes related to pain, GM strength, and functional performance in subjects with CMLBP. It was hypothesized that the Powers’ program, when combined with conventional treatment, would result in greater improvements in GM strength, pain reduction, disability, and functional performance in subjects with CMLBP compared to conventional treatment alone.

## Methods

### Study design

The study was a parallel, single-blinded, randomized controlled trial following the consolidated standards for reporting clinical trials (CONSORT) guidelines [23]. It was conducted at the outpatient clinic of the faculty of physical therapy, Cairo University, Egypt, from July 2023 to March 2024. It was approved by research ethical committee of faculty of physical therapy, Cairo University (No: P.T.REC/012/003955) and was registered on clinical trials.gov on March 16th, 2023 (NCT05770986).

### Participants and recruitment

Potentially eligible consecutive patients were screened for inclusion and exclusion criteria during the evaluation. If they met the inclusion criteria, they were then invited to participate and asked to sign an informed consent form. Participants were referred by orthopedic physicians who had established a diagnosis of CMLBP. Because the trial was done in a controlled clinical setting (non-open access environment), all recommendations were from orthopedic physicians. Upon referral, a qualified physical therapist with at least ten years of experience assessed each participant to ensure eligibility. The diagnosis of CMLBP was established based on mechanical pain characteristics—pain increased by movement or prolonged posture and reduced by rest—with no indication of neurological involvement or systemic disease. Exclusion criteria included traumatic injury in the lower quadrant within the past six months, patients with severe symptoms, and lumbar instability including spondylolisthesis.

### Randomization and blinding

Consented subjects were randomly allocated (1:1 ratio) into either the study group or the control group using computer-generated random numbers (www.randomization.com). The study group received the Powers’ program plus a conventional intervention, and the control group received a conventional intervention only. Group allocation was concealed by an independent researcher until the assignment was revealed. Participants were blinded to group allocation and were informed that they would receive one of two treatment protocols. A physical therapist who was blinded to group allocation and did not participate in the intervention procedures completed the outcome evaluation. To reduce possible assessor bias, standardized testing procedures were performed consistently to all individuals. The physical therapist who delivered the treatment was not blinded to the treatment groups due to the nature of the interventions.

### Outcomes

Assessments were conducted at baseline and after the 4-week intervention. Data was checked for errors. All primary and secondary outcomes were prespecified in the trial registration.

The primary outcomes were back pain and disability. The Arabic Visual Analogue Scale (VAS) was used to quantify the intensity of the current back pain. It is a reliable instrument (intraclass correlation coefficient for test-retest reliability = 0.97 (95% CI = 0.96 to 0,98)) in assessing chronic musculoskeletal pain, including CMLBP. The minimal clinical important difference (MCID) is 2 points on the 10-cm VAS [24]. It has been shown to be highly correlated with a numeric rating scale, with correlations ranging from 0.62 to 0.91 [25]. It is a 10-cm line that is marked at one end with (0) indicating, no pain, and at the other end by (10) indicating the most severe pain experienced. The patient marked the line, and the recorded distance was rounded to the nearest number. For example, a measure of 4.7 cm was rounded to 5 cm.

The Arabic version of Oswestry Disability Index (ODI) was used to quantify back disability. It is a patient-reported questionnaire consisting of 10 questions related to back condition. Each question was answered on a 6-item Likert scale and was scored from 0 indicating minimum degree of difficulty in that activity to 5 indicating maximum degree of difficulty. The validity of Arabic version of ODI is good (*r* = 0.7 on average) and intra-observer reliability was excellent (ICC: 0.99) [26–28]. Participants chose the best answer reflecting their condition in each domain of ODI. In the case of marking more than one statement in each item, the highest score was taken to give a total ODI score between 0 and 50, which is then multiplied by 2 to produce a percentage score. For every question not answered, the denominator was reduced by 5. A score ranging between 0 and 20 indicates minimal disability, 21 and 40 indicates moderate disability, 41 and 60 indicates severe disability, 61 and 80 indicates crippled, 81 and 100 is a bedbound patient [26, 28]. The MCID for back pain and disability of the ODI was reported to be 2.5 and 10 points, respectively [29].

The secondary outcomes were GM strength and functional performance (Single limb triple hop test for distance and Single-Limb Crossover Hop Test for Distance). A Hand-held dynamometer (HHD) (Baseline Pull Push Dynamometer (Model 12–0343, Fabrication Enterprises Inc., NY, USA) was used to measure GM muscle strength. Test-retest reliability Intraclass correlation coefficients (ICCs) of HHD ranged from 0.85 to 0.99 [30]. The patient was positioned prone with knee to be tested flexed to 90 degrees. The researcher put the HHD proximal to the popliteal fossa of the limb to be tested, extended the elbow and asked the patient to lift the thigh off the table on the order of “push,push, push- and relax”. Two trials of 5-second duration, isometric contraction were performed.

In the Single-Limb Triple Hop test for distance (SLTH), The patient’s toes were on the starting line as they stood on one limb. The patient landed on the same limb after completing three consecutive maximal hops as far as possible forward and landed on the same limb. From the starting line to where the patient’s heel landed on the third hop, the distance hopped was measured in centimeters. The patient was given two practice trials and two recorded trials. The test was repeated on the other limb. In the Single-Limb Crossover Hop Test for Distance (SLCHT), the patient performed similar to SLTH but the patient alternatively hopped crossing over a 15-cm strip on the floor. A trial was discarded if an individual touched down with the contralateral limb or did not completely clear the 15-cm width of the line [31].

### Interventions

Subjects in both groups received 12 treatment sessions over four weeks (three sessions per week).

#### Control group: conventional physical therapy intervention

All participants in the control group underwent a standardized conventional intervention protocol comprising flexibility exercises for back and hip muscles, application of a hot pack for 10 min, and transcutaneous electrical stimulation (TENS) using high-frequency parameters (≥ 80 Hz, pulse width < 150 µs), applied for 15 min over the most painful area [10, 32, 33]. This conventional physical therapy is the usual clinical treatment frequently indicated for patients with CMLBP. This multimodal therapy was designed to alleviate discomfort and enhance mobility. A detailed description of the intervention is provided in appendix I.

#### Study group: powers’ Program + Conventional therapy

In addition to the conventional therapy described above, participants in the study group followed the eight phases of the Powers’ program. Phases 1–3 of the program are focused on GM activation. Phases 4–5 are focused on GM strength, and the final three phases emphasize functional applications of the GM during ballistic tasks. Phases 1–5 were performed during the first two weeks. After that, all phases were applied. Detailed exercise descriptions are provided in Appendix I.

Three resistance bands were used through the exercise progression: yellow (low resistance), green (moderate resistance), to blue (high resistance). Three sets of 10 repetitions were performed [21, 22]. Progression through the eight phases of the Powers’ program was contingent upon the participant’s ability to successfully meet the performance criteria for each phase. Participants were expected to complete all specified repetitions (and isometric holds as needed) with the three resistance bands before moving on to the next phase.

### Sample size

**Fig. 1:**
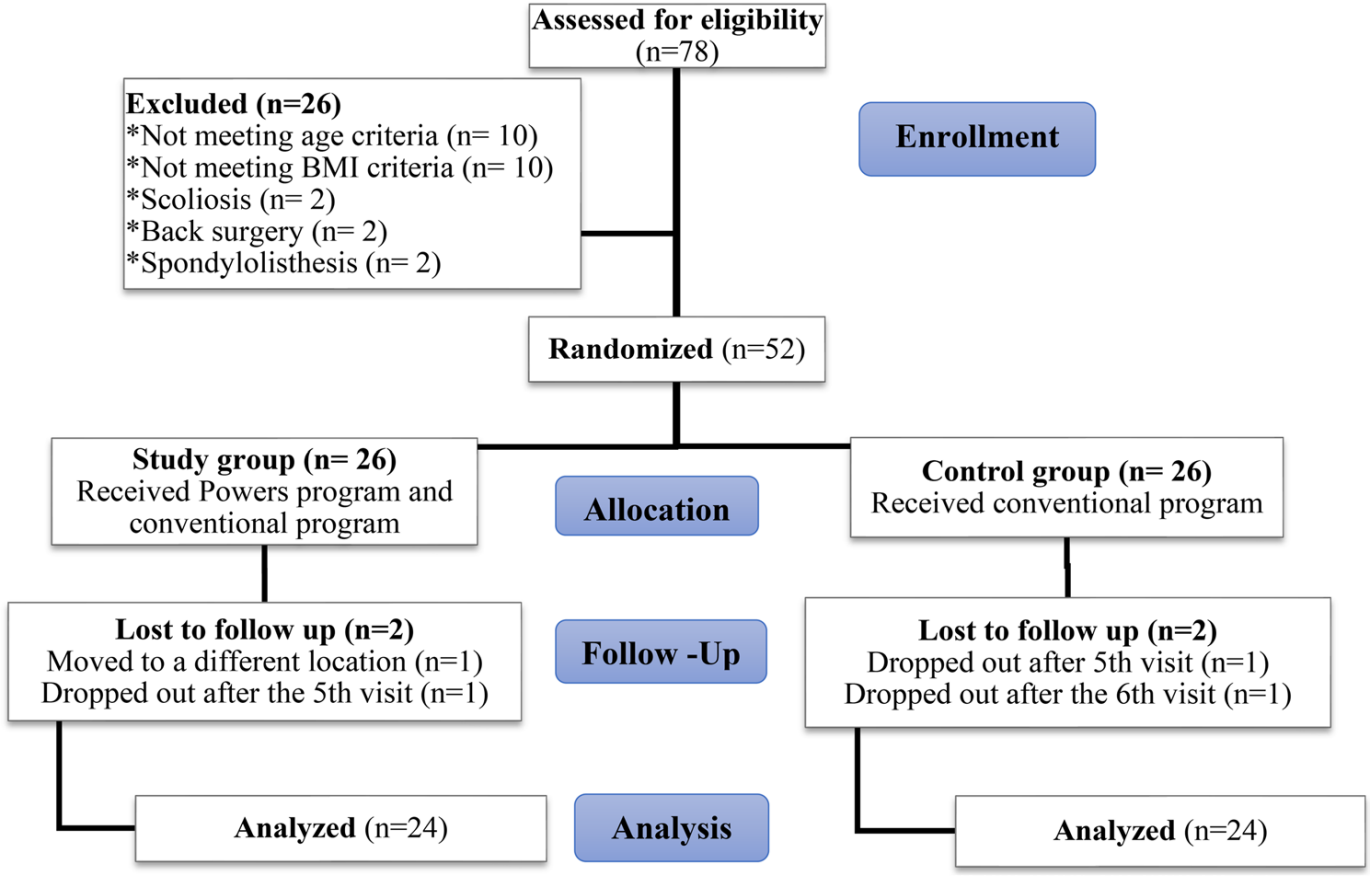
Flow chart illustrating the study progression

A priori power analysis revealed that 52 patients (26 in each group) were needed to detect an effect size of Cohen’s d = 0.80 with 80% power (alpha = 0.05), in an independent sample t-test. G*power software (version 3.1.9.7) was used [34]. There was no interim analysis, and the study was completed without stopping. A flow chart detailing the progression of the clinical trial can be found in Fig. 1. Two participants dropped out from each group and removed from analysis.

### Statistical analysis

Data was analyzed using the statistical package for social science (SPSS) program, version 27 (SPSS Inc, Chicago, IL, USA). Test of normality using Shapiro-Wilk test was performed to measure the distribution of data and showed that data did not violate the assumption of normality. Data were reported as mean ± SD for quantitative variables and frequency distribution (%) for categorical variables. Comparison of the continuous and categorical demographic variables between the two groups was conducted using an independent student t-test and chi-square test, respectively (likelihood ratio was reported if assumption was violated for chi-squared test). Comparison between the mean values of the tested variables between the two groups was conducted using the multivariate analysis of variance (MANOVA) test. Comparison between the mean values of the tested variables between subjects in the same group was conducted using paired student t-test. Alpha level was set at 0.05. No subgroup or sensitivity analysis was performed.

## Results

Baseline characteristics for both groups are shown in Table 1. There were no significant differences in all baseline characteristics between groups (*p* > 0.05) except for the level of activity (*p* = 0.04). (Table 1)

**Table 1:** Comparison of age, BMI, and gender distribution between groups (*N* = 48)

Variables	Study group (*n* = 24)^a^	Control group (*n* = 24) ^a^	Test value^b^	*p*-value
Age (years), $\overline{X}$± SD	32.04 (5.95)	33.50 (6.29)	0.83	0.41
BMI (kg/m²),$\overline{X}$± SD	27.22 (2.10)	27.35 (2.66)	0.19	0.85
Gender, N (%)				
*Male*	3 (12.5)	5 (20.8)	0.6	0.44
*Female*	21 (85.5)	19(79.2)		
Dominance, N (%)				
*Right*	20 (83.3)	31 (87.5)	0.17	0.68
*Left*	4 (16.7)	3 (12.5)		
Occupation				
*Healthcare professionals*	5 (20.8)	6 (25)	0.42	0.81
*Housewife*	11 (45.8)	12 (50)		
*Administrative/clerical*	8 (33.3)	6 (25)		
Level of activity, N (%)				
*Low*	2 (8.3)	7 (29.2)	6.26	0.04^*^
*Medium*	16 (66.7)	8 (33.3)		
*High*	6 (25)	9 (37.5)		
Duration of symptoms, N (%)				
*3 months to 1 year*	9 (37.5)	9 (37.5)	4.82	0.09
*1–3 years*	10 (41.7)	4 (16.7)		
*More than 3 years*	5 (20.8)	11 (45.8)		
Marital status, N (%)				
*Single*	1 (4.2)	1 (4.2)	2.86	0.24
*Married*	21 (87.5)	23 (95.8)		
*Divorced*	2 (8.3)	0 (0)		

*Abbreviations*:$\overline{X}$: Mean, *SD* Standard deviation

^a^Count (percent) otherwise specified, ^b^ test value was reported for continuous variable and chi square/likelihood ration was reported for categorical variables, MD: Mean difference; *p* value: Probability value; ^*^ Significant at *P*<0.05

Scores of the primary (pain and disability) and secondary (GM strength and functional testing) outcomes are presented in Table 2. There were significant improvements in all outcomes in each group post treatment (*p* ≤ 0.001). (Table 2).

**Table 2:** Mean values of pain, disability, GM muscle strength, and functional testing (Hop tests) pre and post treatment of both groups (*N*=48)

Outcomes	Groups		Pre treatment	Post treatment	MD	t- value	p-value
			**Mean ± SD**	**Mean ± SD**			
Pain	**Study**		5.96 ± 1.17	2.67 ± 1.52	3.29	10.06	< 0.001^**^
	**Control**		5.82 ± 0.96	3.44 ± 1.90	2.38	5.67	< 0.001^**^
Disability	**Study group**		15.38 ± 5.37	6.58 ± 3.65	8.79	9.39	< 0.001^**^
	**Control**		14.46 ± 4.13	8.88 ± 4.35	5.58	4.58	< 0.001^**^
GM strength	**Right**	**Study**	72.28 ± 29.34	100.45 ± 38.74	−28.18	−5.83	< 0.001^**^
		**Control**	59.41 (29.87)	80.33 (28.38)	−20.62	−4.30	< 0.001^**^
	**Left**	**Study**	70.03 ± 23.54	98.20 ± 34.51	−28.18	−5.10	< 0.001^**^
		**Control**	54.10 ± 27.66	76.77 ± 24.68	−22.66	−4.98	< 0.001^**^
SLTHT	**Right**	**Study**	200.35 ± 67.49	259.75 ± 63.08	−59.40	−6.94	< 0.001^**^
		**Control**	190.96 ± 54.65	235.04 ± 58.67	−44.08	−6.23	< 0.001^**^
	**Left**	**Study**	191.04 ± 63.78	251.00 ± 67.63	−59.96	−5.61	< 0.001^**^
		**Control**	181.35 ± 56.87	224.63 ± 51.55	−43.27	−4.50	< 0.001^**^
SLCHT	**Right**	**Study group** (*n*** = 24)**	155.25 ± 56.44	212.50 ± 64.12	−57.25	−7.89	< 0.001^**^
		**Control**	142.10 ± 43.15	190.83 ± 53.29	−48.73	−5.41	< 0.001^**^
	**Left**	**Study group**	144.10 ± 49.49	203.42 ± 64.37	−59.31	−7.45	< 0.001^**^
		**Control**	142.88 ± 46.48	183.58 ± 45.87	−40.71	−4.50	< 0.001^**^

*Abbreviations*: *SLTHT* Single limb triple hop test (for distance), *SLCHT* Single limb crossover hop test (for distance), *GM* Gluteus Maximus, *SD* Standard deviation, t- value: t-test value, *MD* Mean difference, *p* value Probability value, ^**^Significant at *P*<0.01

Comparison between the two groups in all outcomes showed a non-statistically significant MANOVA main effect of the grouping variables, with Pillais’ Trace = 0.18, F_(8,39)_ = 1.07, *P* = 0.40. As a follow up test to the MANOVA, a series of one-way ANOVAs on each of the outcomes were performed. As shown in Table 3, none of the ANOVAs were significant (*p* > 0.05) except for GM muscle strength of right and left sides. Since just two groups were compared, no further post-hoc pairwise tests were necessary. However, the results should be taken with caution due to the increased chance of type I error caused by numerous comparisons.

**Table 3:** Tests of between-subject effects (separate ANOVA) for each variable

	Levene’s		ANOVAs- group		
	F _(1,46)_	*P*	F _(1,46)_	*P*	η2
Pain	1.61	0.21	2.36	0.13	0.05
GM strength right	2.17	0.15	4.34	0.04	0.09
GM strength left	3.28	0.08	6.13	0.02	0.12
SLTHT right	0.25	0.62	1.97	0.17	0.04
SLTHT left	1.90	0.18	2.31	0.14	0.05
SLCHT right	0.72	0.40	1.62	0.21	0.03
SLCHT left	3.33	0.08	1.51	0.23	0.03
Disability	0.73	0.40	3.92	0.054	0.08

*Abbreviations*: *SLTHT* Single limb triple hop test, *SLCHT* Single limb crossover hop test, *GM* Gluteus Maximus, F: Fisher value, *P* probability value, η2: partial eta square, significant at *P*<0.05

## Discussion

Adding GM strengthening using Powers’ program to a conventional program showed higher significant improvements in GM strength compared to the conventional program alone. This reflects the cumulative effect of the multi-phase Powers Program, which progressively integrates activation, resistance, and functional exercises. Mean differences (pretreatment minus post-treatment) within study group vs. control group indicated clinical significance of adding Powers’ program as the effects reached the MCID of 2.5 and 10 points for pain and disability of the ODI, respectively [29]. Therefore, clinicians may consider adding Powers’ program to a conventional program of flexibility exercises, hot pack, and transcutaneous electrical stimulation for patients with CMLBP.

The absence of statistically significant between-group differences in pain and disability may be attributed to the influence of the intervention duration. Therefore, differences between the groups may emerge in functional aspects if the experiment was evaluated and conducted over a longer period. Notably, subjects in the study group demonstrated improvements in both pain, GM strengthening, and functional outcomes. These results align with previous studies [6, 7, 13], which reported that weakness of GM decreases spinal stability and controlled mobility and increases spinal loading, which may exacerbate pain.

The higher improvements in GM strength observed in the study group than the control group are likely attributable to the beneficial effect of Powers’ program. These findings corroborate prior systematic reviews indicating that hip muscle strengthening contributes to reduced pain and disability in individuals with low back pain [35]. This multidimensional approach may have facilitated enhanced neuromuscular activation, thereby contributing to the observed improvements in GM strength and, consequently, disability. Additionally, the structured and progressive nature of Powers’ program may have increased patient adherence and engagement through sustained therapist interaction [35].

The importance of supplementing core stabilization with additional exercise modalities is further supported by a recent study by Kuzu et al. [36], which demonstrated that adding aerobic exercise to core stabilization in geriatric individuals with chronic non-specific low back pain led to superior improvements in functional capacity and physical performance compared to core exercises alone. This supports the inclusion of GM-focused exercises in rehabilitation programs targeting CMLBP.

The finding of the current study agrees with Lee [37] who found that novel and standard gluteal strengthening programs are equally effective in improving pain and functional disability. However, the result of the present study differs from Lee’s in terms of gluteal muscle strength difference between groups. While Lee found no significant difference between the two programs, the present study demonstrated that the study group experienced greater improvements in gluteal muscle strength [38].

Consistent with the findings of Fukuda et al. [39], the present study did not find statistically significant between-group differences in pain or disability following the addition of hip strengthening to conventional therapy. One plausible explanation, as suggested by prior research [39], is that individuals with low back pain may exhibit elevated baseline GM activation, limiting the marginal benefit of additional activation-focused exercises on pain outcomes. Nonetheless, the observed improvements in GM strength were significantly greater in the study group and may have contributed to the greater, albeit non-significant, improvements in disability. These findings suggest a closer relationship between GM strength and functional disability than with pain reduction.

Although the study hypothesized that the Powers Program would improve GM strength and thus reduce pain and disability, the results showed that muscle strength improvements did not lead to better clinical outcomes in pain or function than the conventional program. These findings indicate that, while focused hip strengthening may improve local muscle function, pain and disability in CMLBP are likely driven by variables other than muscle strength alone.

Moreover, this study did not involve direct biomechanical assessments, but the reported increases in GM strength and functional performance are likely due to positive mechanical adaptations. The GM is responsible for controlling hip extension and lumbopelvic stability; hence, strengthening this muscle may indirectly improve mechanical load transfer and movement efficiency in those with CMLBP. While the functional hop tests utilized in this study were not direct biomechanical measures, they did involve coordinated lower-limb and trunk control, indicating that mechanical integration improved with training. These findings support the theoretical logic that focused GM strengthening helps to restore normal lumbopelvic rhythm while also lowering compensatory stress of the lumbar spine and sacroiliac joint—mechanical components frequently involved in CMLBP. Future research may use kinematic or kinetic analysis to measure these changes.

To the best of the authors’ knowledge, this study represents the first clinical investigation evaluating the impact of Powers’ program on patients with CMLBP. Nevertheless, this study is limited by small sample size, the absence of a true control group, and the restriction of the sample to young adults. Therefore, the generalizability of the results must be taken with caution. Furthermore, treatment protocols were not individualized based on specific patient impairments, and potential biases may have been introduced by clinician and patient expectations. Because multiple univariate ANOVAs were inspected following the MANOVA, there remains a possibility of type I error inflation, although no additional post-hoc tests were performed.

Moreover, despite random assignment, there was a substantial variation in baseline activity levels between groups (*p* = 0.04). This discrepancy might have functioned as a confounding variable, altering functional performance results. This imbalance was most likely caused by small sample size, which should be taken into account when interpreting the results. Finally, The overall exercise intensity and duration may not have been the same across the intervention and control groups. As a result, the observed increase in GM strength might be attributed to a greater total exercise dosage rather than the Powers program’s unique benefits. Future research could include matching exercise dose or incorporating a placebo exercise group to identify intervention-specific effects.

## Conclusion

Addition of Powers’ program to the conventional intervention provided higher improvement in GM strength and disability, compared to the conventional intervention alone in patients with CMLBP, indicating consistency with previous literature that addition of hip strength is effective for CMLBP. However, further research is needed to support this using a larger sample size and longer treatment duration and to determine which specific patients benefited most of the Powers’ program.

## **K**ey **P**oints

### Findings

Addition of Powers’ program to the conventional physical therapy intervention provided higher improvement in GM strength and disability, compared to the conventional program alone in patients with CMLBP.

### Implications

This clinical trial supports the addition of Powers’ program to the conventional physical therapy intervention in treating patients with CMLBP.

## Supplementary Information

Supplementary Material 1

## Data Availability

The datasets used and/or analyzed during the current study are available from the corresponding author on a reasonable request.
