# Supplementary material for: Impact of gluteus maximus–focused intervention using Powers’ program on chronic mechanical low back pain: A randomized controlled trial
Source: BMC Sports Sci Med Rehabil. 2026 Jan 20;18:84. doi: 10.1186/s13102-025-01475-x (PMC12903588; doi:10.1186/s13102-025-01475-x)
Supplement: Supplementary file 1 — Supplementary Material 1 [file 13102_2025_1475_MOESM1_ESM.docx]

**APPENDIX I**

**Summary of the intervention**

| **Conventional program** | **Powers^,^ program** |
| --- | --- |
| **Flexibility exercises of the back muscles:** knee to chest stretch, bridging, stretching exercises for hip flexors, piriformis, adductors and hamstring will be performed, with a hold of position for 30 s and with repetitions (3–5 times). Static stretches will be held for 30 seconds to induce changes in flexibility and will be repeated for three sets. Three sessions of stretching per week will be performed.^10^  **Hot pack:** for 10 minutes.^23^  **TENS:** High frequency or conventional TENS (frequency greater than 80 Hz, pulse width less than 150 microseconds (μsec) was used. ^24^ The electrodes were placed over the area of most severe pain for 15 minutes. The intensity was gradually increased until the sensation feels strong but comfortabl.^24^ | **Phase 1:**  **1. Activation- Clam (clamshell):** The patient was in a side lying position, with the angle of the hip flexion less than 20 degree to prevent the firing of tensor fascia lata (TFL). A loop green band resistance was around the patient’s distal thigh. The patient was asked to spread the knees apart keeping the feet together and hold. The progression of exercise to increase hold time to maximum level (one minute for 5 times with blue band).^21,22^  **2. Activation- Fire Hydrant:** The patient started supporting his/her elbows on the table. Patient was instructed to tighten his core muscles and lift his/her leg into extension, abduction, external rotation against the loop resistance band, then to lower his leg to the starting position. The knee was kept at 90 degrees of flexion. ^21,22^  **Phase 2:**  **1. Activation- Squat:** A loop resistance band was placed above patients’ knees. Patient stood with the feet slightly wider than shoulder, toes pointed slightly out, and hands on hips or in front. Then, the patient hinged at the hips and pushed the buttock back into a sitting position while bending knees slightly while resisting the band. The patient continued to lower him/herself until his/her thighs were parallel to the floor or lower. The patient slowly lifted back up into the starting position by pushing into the heels and squeezing the glutes.^21^  **2. Activation- Surfer:** A loop resistance band was placed above patients’ knees. Patient stood with his/her feet slightly wider than shoulder, toes pointed slightly out. The patient performed a partial squat against the loop resistance band while rotating one leg out into external rotation, at the same time twisted the trunk and point the hands to the side of trunk twist. The patient then returned to the starting position.  **Phase 3:**  **Activation- Standing Fire Hydrant:** A band was placed above patient knees while standing then he/she was instructed to tighten the core muscles and lift leg into extension, abduction, external rotation against the loop resistance band, hold for 30 seconds 5 times, then lower the leg to the starting position. The knee was kept at 90 degrees of flexion.^21^  **Phase 4:**  **1. Strength- Crab Walk**: The patient assumed a squat position of approximately 45° of hip and knee flexion. Thera-Band was positioned at the level of the mid-thigh to resist bilateral hip abduction and external rotation. The patient was asked to step to the right and left along a 10-m walkway by abducting and externally rotating the hips, with feet shoulder width apart for each step for 2 sets of 15 steps.^21^  **2. Strength- Lunge:** The patient started in a standing position with the feet hip-width apart. The patient was instructed to step forward longer than a walking stride so one leg is ahead of the torso and the other is behind. The foot should land flat and remain flat while it is on the ground. The rear heel rose off of the ground. The patient bent the knees to approximately 90 degrees as lowering him/herself. Instruction was given to keep trunk upright and [core engaged](https://www.healthline.com/nutrition/how-to-engage-your-core). Then, forcefully he/she pushed off from the front leg to return to the starting position. No resistance band was used for this exercise.^1^  **Phase 5:**  **1. Strength- Split Squat**: Patient started by standing about 2 feet in front of a knee-level bench or step then lifted the right leg up behind him/her and place the top of his/her foot on the bench. The feet were about shoulder-width apart, and his/her right foot was far enough in front of the bench where he/she comfortably lunged and hopped around a bit. The patient rolled his/her shoulders back and leaned slightly forward at the waist, began to lower down on his/her left leg, bent the knee.  **2. Strength- Romanian Dead Lift:** Patients balanced on their dominant limb, with their knee and hip flexed approximately 30° and their hands on their hips. Patients slowly flexed their hip and trunk and touched their contralateral middle finger to the ground beside their support foot and returned to the starting position. Patients were instructed to keep their knee flexed 30° when reaching for the desired level.  **3. Strength- Single Leg Squat:** Patients started the squat by balancing on their dominant lower extremity, with their knee and hip flexed approximately 30°. Patients slowly lowered themselves toward the ground, using their ankle, knee, and hip joints. Patients then returned to the starting position and were instructed to keep their knees over their toes to prevent a knee valgus position.^21^  **Phase 6:**  **1. Forward Jump**: Patient stood with his/her feet directly under his hips and arms alongside his/her body. He/she drew his/her shoulder blades back and down. Then, the patient dropped his hips back and down into a squat position. He/she kept his/her elbows straight while extending his/her arms behind. He/she jumped forward, pushing with his/her feet and straightening his/her legs. At the same time, he extended his/her arms overhead. The patient pulled his/her legs forward as he/she lands. To reduce the impact, he/she bent the knees and hinged the hips slightly forward, lowering into a squat position.^21^  **2. Lateral jump:** Patient started with his/her feet no more than hip-width apart, he/she was instructed to bend his/her knees to [squat](https://www.verywellfit.com/safe-squat-technique-3119136) straight down. Then he shifted weight from heels to toes as he/she begins his jump, quickly he/she pushed upward and sideways toward the other side of the line. The patient was instructed to land softly and absorb the shock by squatting deeply. He/she repeated jumping back and forth over the line while keeping his/her shoulders and hips square and facing forward. Instruction was given to keep knees aligned and for soft landings.  **Phase 7:**  **Single leg forward hop:** Patient stood with his/her feet at shoulder width apart and lifted his/her left leg off the floor, pointing it behind him. Then, he/she bent his/her right knee slightly and then pushed through the floor explosively to allow the body to hop from the ground. As he/she lands, He/she was instructed to be cushion the impact by once again bending his/her right knee. Three sets of five repetitions were performed before changing sides.^21^  **Phase 8:**  **Cutting (Zig-zag jump):** Cones were placed about 3 feet apart from each other in a zig zag pattern. One cone was placed in front of the patient, then the next cone about 3 feet to the right in a diagonal. The next cone went about three feet to the left in a diagonal, and so on. Patient started standing in front of the first cone at the base of the zig zag formation then bent his/her knees and jumped forward diagonally to the second cone. At the second cone, the patient was instructed to land softly with knees bent to prepare for the next jump. He/she leaned forward and jumped diagonally and to the left towards the third cone. The patient continued jumping in a diagonal pattern from cone to cone until he/she came to the last cone. Finally, he/she turned around and returned to the starting position. |
